# Using places of worship to recruit and retain couples for the ‘Diabetes Together’ intervention

**DOI:** 10.4102/phcfm.v17i1.4947

**Published:** 2025-07-21

**Authors:** Cathryn Pinto, Thandi Puoane, Darcelle Schouw, Buyelwa Majikela-Dlangamandla, Cynthia Paka, Kenneth Muhali, Ishaaq Datay, Peter Delobelle, Naomi Levitt, Nuala McGrath

**Affiliations:** 1 Department of Primary Care, Population Sciences, and Medical Education, Faculty of Medicine, University of Southampton, Southampton, United Kingdom; 2 School of Public Health, University of the Western Cape, Cape Town, South Africa; 3 Department of Family Medicine and Emergency Medicine, Stellenbosch University, Cape Town, South Africa; 4 Chronic Diseases Initiative for Africa, Faculty of Medicine, University of Cape Town, Cape Town, South Africa; 5 Department of Public Health, Vrije Universiteit Brussel, Brussels, Belgium; 6 Department of Social Statistics and Demography, Faculty of Social Sciences, University of Southampton, Southampton, United Kingdom; 7 School of Nursing and Public Health, University of KwaZulu-Natal, Durban, South Africa

**Keywords:** faith-based settings, churches, recruitment, intervention, couples, Type 2 diabetes, South Africa

## Abstract

**Background:**

There is a growing prevalence of type 2 diabetes (T2D) in South Africa and a high proportion of people have poor glycaemic control.

**Aim:**

Having developed ‘Diabetes Together’, a couples-based intervention to support diabetes self-management, we explored places of worship as potential options for recruiting couples in the community.

**Setting:**

Places of worship in low-income settings in Cape Town, South Africa.

**Methods:**

Community entry involved approaching leadership of each place of worship to discuss the programme and our target of recruiting 15–20 eligible couples, where one partner was living with T2D. The research team and study were introduced to each congregation. Enrolment took place at the first of three intervention sessions. Attendance, participant feedback and facilitator observations were recorded. Recruitment and retention outcomes were summarised using descriptive statistics. Participant and facilitator feedback was deductively coded based on the evaluation questions and overarching themes identified.

**Results:**

The intervention was conducted in two churches and one mosque after engaging with leaders of six places of worship. A total of 37 people living with T2D were screened; 34 were eligible and had a self-reported T2D diagnosis, 32 partners were screened. Twenty-nine couples were eligible, and 24 couples enrolled. Retention was good across all three places, minimum 75% by session three. Participant and facilitator feedback revealed that participants gained new knowledge, reported having a positive attitude towards diabetes management and valued group interaction and open communication.

**Conclusion:**

Recruitment of couples from places of worship in low-income settings in Cape Town was feasible under certain conditions. The intervention was acceptable and retention of couples for repeated sessions was high.

**Contribution:**

As there is limited evidence on using community settings like places of worship for diabetes management programmes, we present practical considerations for successful recruitment from these settings in South Africa.

## Introduction

Sub-Saharan Africa (SSA) is facing a growing prevalence of type 2 diabetes (T2D), with a high proportion of people living with type 2 diabetes (PLWD) having poor glycaemic control.^1,2^

Healthcare systems in SSA are unable to cope with the current burden of T2D and its complications and more effective educational and self-management interventions are needed.^3^ A systematic review found poor adherence to T2D self-management behaviours such as glucose monitoring, physical activity, diet and medication.^4^ In SSA, therapeutic patient education programmes have a positive impact on clinical outcomes (e.g. blood glucose levels, body mass index, blood pressure and lipid profiles) and on improving knowledge and self-management skills.^5^ However, the sustainability of these programmes and the lasting effects on self-management behaviours is uncertain.

One way to improve long-term behaviour change is to consider the family context which can act as a source of motivation and support for PLWD. Studies have shown that couples-based interventions can be effective at improving self-management for people living with long-term conditions,^6^ and studies in high-income countries offer encouraging evidence for the benefits of couples-based interventions with respect to managing T2D.^7,8,9,10,11^ Our research team developed a couples-based intervention to promote T2D self-management called ‘Diabetes Together’ for South Africa. The intervention was tailored to couples in low-income communities in Cape Town where one partner was living with T2D by using the Person-based approach and drawing on existing evidence, primary interviews with target users and a process evaluation of pilot intervention delivery to 14 couples. The ‘Diabetes Together’ programme involved two group workshops, plus individual support sessions. The first workshop was conducted for the whole group together, and in the second workshop, participants were split into subgroups based on their gender and their diabetes status.

Having completed a process evaluation, we plan to conduct an effectiveness trial of the Diabetes Together intervention, but the method of recruitment for the trial is unclear. Our previous experience included approaching PLWD attending geographically dispersed primary care diabetes clinics and using snowball sampling methods during coronavirus disease (COVID-19).^12^ Clinic spaces are busy providing routine care and COVID-19 restrictions were in place, which made it difficult to recruit a large sample from any individual clinic. Participants were requested to travel to a central location to take part in the intervention group sessions, which could have been an additional barrier. Our intervention is community-oriented and in a future trial, we wish to deliver it within communities close to where we recruit eligible couples.

One potential site is places of worship; affiliation to places of worship is common in South Africa. In 2018, 75% of people in South Africa who were surveyed said religion is important to them and 55% regularly attend places of worship with the majority describing their religious affiliation as Christian (84%), followed by traditional African religions (5%) and Muslim (2%).^13,14^ In the US, health promotion programmes, including diabetes prevention programmes in churches were effective for promoting behaviours such as healthy eating, physical activity and disease screening, including programmes with African–American communities.^15,16,17^ In South Africa, a healthy lifestyle programme was delivered through churches and was found to be effective in improving health outcomes such as weight loss and blood pressure among participants.^18^ However, there is limited evidence for targeting communities attending places of worship for diabetes prevention and management in South Africa.

There has not been any research on couples-based interventions for diabetes management in faith-based settings in South Africa. We hypothesised that places of worship in low-income communities would be useful for identifying and retaining couples where one partner has T2D because places of worship are likely to be attended regularly, and delivering the intervention in a location that participants were familiar with would support ongoing engagement with the intervention and study follow-up. The aim of this study was to test the feasibility of recruitment and retention of eligible couples and intervention acceptability for the Diabetes Together intervention by targeting communities attending places of worship. We wanted to purposively sample different places of worship, including different Christian denominations, and within the limited study period, deliver the intervention in three places of worship sequentially to enable learning from one context before moving to the next. The aim was also to target communities likely to receive healthcare from public clinics, and for this purpose, the places of worship were selected accordingly.

## Research methods and design

### Study design

This is a mixed-methods study assessing the feasibility of recruitment and retention and the acceptability of the Diabetes Together intervention in the places of worship. A prospective cohort was enrolled in each place with retention as the outcome.

### Setting

The study was conducted among members attending places of worship in three low-income peri-urban settings in Cape Town, South Africa.

### Study population and sampling strategy

Convenience sampling was used to select places of worship based on the existing networks and contacts of research team members. Outreach activities involved contacting the leader of the place of worship, explaining the study and process, asking for support to conduct the research, permission to recruit participants and field visits to prospective venues to be used for intervention delivery, agreed in partnership with the leadership at the place of worship. Members of the research team attended a service at each potential place of worship to assess the size and composition of the congregation. The research team were formally introduced to the congregation at a service by the leadership and were present to answer questions, screen participants or collect prospective participants’ contact details. Copies of the study advert were available either for potential participants, their partners or for someone they knew who might be interested in taking part in the study. The introductions were performed at least 2 weeks before the first intervention session to allow for screening. Eligible couples were invited to the first session, which was scheduled on a date predetermined with the leadership of the place of worship.

The target for enrolment was 15–20 couples at each place of worship as this allowed for ease of facilitation and for participation in interactive tasks in a group setting. We intended to deliver each session once, and from previous experience, we identified this as an appropriate number of couples to invite for the sessions allowing for potential drop out. The study’s introduction and study advert clearly stated the eligibility criteria for taking part in the study. This tended to be those who came forward and expressed an interest in taking part. We screened all PLWD who came forward based on their eligibility criteria. Eligible PLWD included people aged 18 years or above, living with T2D who attended a public sector clinic for routine healthcare and were involved in a partner relationship for at least six months. People living with T2D, who received healthcare from a private clinic or who were not in a relationship with a partner were excluded. Their partners included people aged 18 years or above and not living with T2D, because the aim of intervention was for partners to support diabetes self-management for the PLWD, by equalising diabetes knowledge between partners and developing a shared appraisal and joint action towards diabetes management. Participants needed to speak and/or have a minimal understanding of the English language to be able to engage effectively with the intervention material which was assessed during screening. Individuals who gave the research team their contact details at the place of worship or who read the study advert and contacted the research team afterwards, were screened in person after the study introduction or by phone using a screening tool (Online Appendix 1). People living with T2D were screened first, followed by their partners, if they were eligible. All eligible couples were invited to the first group session at their place of worship and each participant was sent a copy of the information sheet and consent form in print or *via* WhatsApp in their preferred language. Details were discussed over the phone with a researcher to facilitate providing informed consent and enrolling in person at the first group session. Each member of the eligible couple received two reminders *via* WhatsApp a few days before the first group session. Only couples who attended the first session and completed the consent form were enrolled. Couples needed to attend the sessions together and each member of the couple received a grocery store voucher of ZAR150 ($8.15 as of June 2024) after every session they attended as reimbursement for their time, inconvenience and expenses.

### Intervention

The intervention was delivered in group sessions on three consecutive weekends at a conveniently located venue close to the respective places of worship, each session lasting for 2 hours. The venues for the delivery of the intervention sessions were selected in discussion with the leadership as they were safe and accessible spaces. Four selected modules from the Diabetes Together intervention (i.e. Diabetes Key information, Couples Communication, Getting active, Eat for health) were delivered to participants together as a couple.^19^ The sessions were delivered by two facilitators who were registered nurses (Cy.P. and B.M.D., who were also involved in the development and optimisation of the Diabetes Together programme) and supported by two researchers who have previously worked in delivering diabetes management programmes (K.M., D.S.). The sessions involved a combination of informational videos and interactive group activities and discussions facilitated by the research team. Handouts were given to participants at the end of each session as a reminder of the content covered. To ensure intervention fidelity and quality control across different places of worship, training was provided to facilitators on the intervention content and research procedures. The intervention followed a set script for each module and a set agenda for each session. Facilitators completed a report at the end of each session and were in regular contact with the research co-ordinator C. Pinto and the wider team. Additionally, an in-person site visit was carried out by C. Pinto to monitor intervention delivery in one of the places of worship.

### Data collection

Feasibility was assessed by determining initial interest from the leadership and congregation, recruitment and enrolment rates, retention through intervention session attendance rates, and facilitator reflections on challenges and barriers to implementation. Acceptability of the intervention sessions was assessed through participant feedback and facilitator observations. The screening survey captured demographic characteristics of individuals showing interest in taking part and their eligibility status after screening. The research team recorded their observations during the introduction session at each place of worship. Participant attendance was recorded for the group sessions to measure retention. Participants completed evaluation forms at the end of each session and facilitators and researchers completed debrief forms with observations and reflections after each session (see Online Appendix 2 and Online Appendix 3).

### Data analysis

Participants’ characteristics, recruitment and retention at each place of worship were analysed and contrasted using descriptive statistics. Feedback from the participant and facilitator evaluation forms were coded deductively following a framework guided by the evaluation questions and the session content. The codes were discussed with the research team to identify patterns across the data and to ensure trustworthiness in the analysis. Themes were identified across both participant and facilitator feedback codes and illustrative quotes were provided to demonstrate credibility. Research team observations and reflections around community outreach and intervention delivery were summarised and described to give context to the findings (see **Appendix 4**).

### Ethical considerations

This study was conducted in accordance with the Declaration of Helsinki and received ethical approval from the University of Southampton, Faculty of Medicine Ethics Committee (No. ERGO 53875.A4) and the University of Cape Town (Human Research Ethics Committee (No. 031/2020). All participants gave informed written consent to participate in the study.

## Results

### Community outreach and study introduction

Six different places of worship were approached (four churches – Roman Catholic, World Harvest Christian Centre, Pentecostal, Seventh Day Adventist; one masjid [mosque], and one Muslim women’s group linked with another masjid) and the intervention was delivered in two churches and one masjid between June and October 2024 (see Table 1 and Table 2). The intervention was not delivered in three places of worship (two churches and the Muslim women’s group). For one of these churches, it was not possible to schedule any sessions within the study timeframe because of the congregation experiencing a community loss. At the second church, an educational talk about diabetes was given, but the research team and church leadership concluded that because of the small congregation size, it would not be possible to recruit the target amount (only three couples expressed an interest in taking part). In addition to recruiting at the masjid (predominantly male attendance at services), a Muslim women’s group was also approached, but had recently received a lot of health messaging and were less receptive to receiving the intervention within the study period. We learned several lessons from our screening and recruitment efforts and applied these learnings to subsequent places of worship. At the first place of worship, we scheduled more introduction sessions and planned to mainly screen people after the service at the church. This strategy was less successful and in subsequent places of worship we took down the contact details of interested people and followed up by phone. We also broadened our study promotion activities to include reaching out to people *via* social media and requested the church leadership to actively identify potential participants. Our reflections and lessons learned have been detailed in Online Appendix 4.

**TABLE 1:** An overview of outreach and engagement activities at participating places of worship.

Site	Activities before introduction sessions	Number of introduction sessions	Activities at introduction sessions	Additional ways the study was promoted
Place of worship 1 (Roman Catholic church)	Initial meeting arranged with the priest and two members of the research team.	Two introductions at the main church One introduction at a satellite church	Team was formally introduced during the service, addressed the congregation and provided study details. At the end of the service, the team remained present to register and screen people showing interest.	Shared within church group through a church member that the research team knew previously.
Place of worship 2 (Masjid)	Potential introduction dates and best ways to introduce the study before the Jumu’ah (Friday mid-day congregational prayers) were discussed and agreed upon. Two team members carried out a field visit to determine the logistics and appropriateness of the venue for the intervention sessions.	Two introductions	At a regular Friday service (Jumu’ah), a male research team member (I.D.) gave a culturally adapted pre-Khutbah (talk in a locally understood language) sermon at the starting of prayers, introducing the study and emphasizing how contributing to and co-producing knowledge by enrolling in research studies aligns with the Muslim faith and the Quran. Further study information was provided and a list of those interested was recorded at the main entrance (men) and the entrance for women of the masjid.	Shared on social media and WhatsApp groups linked to the particular masjid.
Place of worship 3 (Pentecostal church)	Dates for intervention sessions were identified in discussion with the bishop and with reference to scheduled events at the church. Two team members conducted a scoping visit to determine the accessibility and venue suitability for the group sessions.	One introduction at main church	Team was formally introduced during the service, addressed the congregation and provided study details. At the end of the service, the team remained present to record contact details of those interested in taking part.	Bishop helped identify eligible couples in the congregation and shared information with them. Study information was shared on social media channels. Bishop contacted linked satellite churches and shared the study information with them and their congregations and invited interested couples to attend the planned introduction.

**TABLE 2:** Characteristics of the population at participating places of worship.

Site	Estimation of congregation size at the service	Estimation of gender split
Place of worship 1 (Roman Catholic church)	60	75% women, 25% men
Place of worship 2 (Masjid)	300	7% women, 93% men
Place of worship 3 (Pentecostal church)	80	50% women, 50% men

### Participants’ screening and recruitment

**FIGURE 1:**
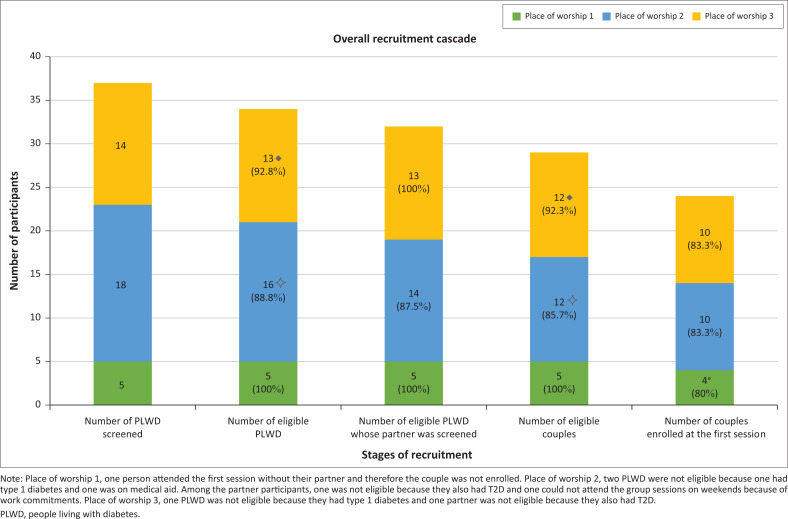
Recruitment flow across three places of worship.

The overall flow of recruitment in the three places of worship where the intervention was delivered is presented in Figure 1. Thirty-seven PLWD were screened, which ultimately yielded 24 couples being enrolled. In addition to those screened at the place of worship 1, 12 PLWD showed interest but were not screened because they did not have a partner, and two people showed interest on behalf of a family member with T2D, but their family member did not contact us. At the place of worship 2, six PLWD did not respond to our calls to schedule screening and four people gave contact details on behalf of a family member but did not respond to our calls (and their family member did not contact us). At the place of worship 3, two PLWD who provided their details did not have partners and two did not respond to calls. Those couples who were screened and eligible were successfully enrolled if both attended the first group session and gave written consent to take part.

Characteristics of study participants are presented in Table 3. The sample included more men who were living with type 2 diabetes and with more women partners. The majority of our sample, both PLWD and their partners, were also living with at least one other long-term condition.

**TABLE 3:** Characteristics of study participants (*N* = 24).

Demographic and clinical characteristics	Person living with diabetes		Partner	
	*n*	%	*n*	%
**Gender**				
Male	15	62.5	9	37.5
Female	9	37.5	15	62.5
**Age (years)**				
40–49	5	20.8	5	20.8
50–59	6	25.0	7	29.0
60–69	11	45.8	8	33.3
70–79	2	8.3	4	16.6
**Preferred language**				
isiXhosa	1	4.1	1	4.1
Afrikaans	3	12.5	3	12.5
English	14	58.3	10	41.6
isiXhosaand English	2	8.3	2	8.3
Afrikaans and English	4	16.6	8	33.3
**Highest education level achieved**				
Primary school	3	12.5	3	12.5
Secondary school	17	70.8	17	70.8
Tertiary education	4	16.6	4	16.6
**Employment**				
Retired	8	33.3	8	33.3
Self-employed	5	20.8	2	8.3
Unemployed	5	20.8	7	29.0
Employed (part-time or full-time)	6	25.0	7	29.0
**Other long-term conditions**				
None	8	33.3	9	37.5
Hypertension	16	66.6	11	45.8
High cholesterol	5	20.8	4	16.6
Other illnesses	5	20.8	9	37.5

### Retention and intervention evaluation

**FIGURE 2:**
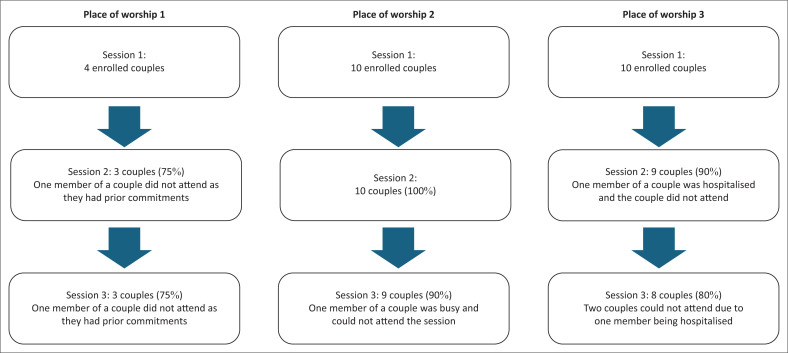
Enrolled couple attendance at follow-up sessions across the three places of worship.

Once participants were enrolled, retention was high for the follow-up sessions across all three places of worship (Figure 2).

#### Participant and facilitator feedback from the intervention sessions

Participant feedback was positive. All participants who completed the feedback questionnaires reported that they were very satisfied with all the three sessions and that they would recommend the sessions to other couples who were interested in T2D management support. Detailed feedback for each session is presented in Online Appendix 5. Three main themes were generated from participants’ and facilitators’ feedback. There were no substantial differences between feedback from participant men and women, or between PLWD and their partners.

#### Theme 1: Gaining new knowledge and change in attitude regarding type 2 diabetes management

Participants reported gaining more knowledge about T2D compared to what they knew before attending the sessions, with a better understanding of how to manage T2D with medication, diet, physical activity, communication and working together as a couple. Their learning mirrored the information provided by each session and the messages conveyed by the intervention material delivered in that session:

‘After 16 years of having diabetes there is a lot I learned today. I learned about what insulin is and what it does.’ (PLWD, female, age 64)

‘I have learned so much from the past week and this session. For example, how to plan your eating habits, your intake, how to control your diabetic through exercise and how to help your partner that has diabetes stay healthy, eat correct and reduce your sugar level.’ (Partner, female, age 60)

Facilitators noticed that during the first session, participants showed varying levels of knowledge about T2D and ways of managing it and they needed to be equipped to answer technical questions around how insulin and medication work. Facilitators observed that all groups actively engaged in activities around identifying healthy food, portion sizes and addressing food myths. There were many questions related to specific foods, the best time to eat during the day and in relation to taking medication or exercising.

Both partners and PLWD reported that they were satisfied with the information provided and they appreciated the knowledge and passion that came through from the facilitators. This information and approach led to participants having more hope that they could take steps to manage their T2D and that it was not up to fate but something that could be controlled and managed:

‘[*I learned*] that it is not too late to make those important physical changes in my life.’ (PLWD, male, age 72)

‘To take my health more seriously and make time for myself.’ (PLWD, female, age 57)

#### Theme 2: Group interaction, sharing experiences and open communication between partners

Participants valued the interaction between group members and learning from each other’s experiences. They liked the educational content that was presented to them through interactive tasks:

‘The group was very good and honest about their experiences of living with a diabetic partner.’ (Partner, female, age 60)

‘The interaction with the group. You learn more from what other people are saying.’ (PLWD, male, age 49)

Both partners and PLWD spoke about the importance of communicating openly as a couple and that talking about communication openly during the sessions and hearing from other couples encouraged them to see their role in the partnership in different and more supportive ways:

‘To communicate properly in order to understand each other. To be able to work out a way to help the person with diabetes.’ (Partner, male, age 70)

‘The talking amongst the couples. Very open information sharing from other couples is good to hear.’ (Partner, male, age 50)

Facilitators also observed that participants were more engaged with session content such as the role plays and identifying healthy and unhealthy foods because it sparked further discussion within the group. They noted a tendency for participants to compare experiences and share tips within the group, prompted by the diabetes information content, although facilitators needed to remind participants that individual symptoms and treatment may differ.

#### Adding more sessions and content

When asked how to improve each session, a few participants highlighted that the sessions were too short and suggested having more sessions or increasing their duration. Some participants wanted additional content about medication, other co-morbid illnesses, examples of exercises (especially chair-based or easy exercises, if experiencing issues with mobility or pain), examples of meal plans, and how to deal with intimacy problems within couples. Facilitators reported that at the end of the sessions, participants approached them with questions around specific medications, natural remedies, dealing with sexual intimacy and alternative exercises because of problems with their feet:

‘I think three sessions isn’t enough to learn everything.’ (PLWD, male, age 65)

‘To maybe give more information on intimacy and how to combat it if you have problems.’ (Partner, male, age 59)

Participants, both PLWD and partners, reported the value of having these sessions on a more regular basis. They also referred to other people in the community who could benefit from these sessions. Facilitators noticed that participants wanted to continue sharing learning beyond the sessions and in place of worship 3, one of the facilitators created a WhatsApp group so that participants could continue to support each other.

## Discussion

Our findings show an appetite for T2D education in places of worship. While it was feasible to recruit couples and deliver a couples-based intervention with good participant retention, the aim of recruiting 15–20 couples in each place of worship was not achieved. The pool of participants at each place of worship, despite two places having a large congregation size or links with satellite venues, may not be sufficient to merit the resource-intensive steps of engagement if the aim is to repeatedly deliver the intervention to groups of 15–20 couples at a time as part of a large randomised controlled trial (RCT). However, the yield of enrolled couples from interested individuals was high compared to other studies that have recruited couple participants.^20^ This may be because partners of interested individuals were more likely to respond to a study invitation that was extended by their place of worship or congregation.

Places of worship are potentially useful sources to identify individual participants in the community who need T2D informational support as shown by the interest from the leadership at the places of worship and from the people who approached the research team after the study introduction. Places of worship may also be particularly good places to reach underserved communities or those who do not regularly access healthcare.^21^ In terms of the feasibility of recruiting couples for an RCT from different places of worship, a lot of time and effort was invested in outreach activities and study introductions, and it was difficult to recruit our target sample size despite broadening our recruitment strategies. We would advise looking at the congregation size to assess whether the potential participant pool is large enough, doing some preparation work with the leadership to link with satellite venues, and making a prior estimation of the likely number of participants from the congregation before proceeding with recruitment and intervention delivery. We also recommend allocating resources (e.g., staff and time) to engage in outreach activities which are often best scheduled on weekends and being sufficiently flexible in terms of timescales for intervention delivery to fit into the existing schedules at the places of worship. In addition, having the leadership endorse the study and the congregation becoming more familiar with the research team can aid recruitment. Working within our recommendations, places of worship can be a feasible option for recruiting participants to a large RCT. In order to optimise and scale up recruitment for an RCT we would recommend exploring other avenues in the community such as health promotion or screening camps, wellness hubs, or use public health messaging through various media channels. These community avenues can complement recruitment *via* health clinics and hospitals to achieve the required target numbers.

With respect to couple retention, reasons for attrition in this study included clashes in schedules where one partner was unable to attend and other personal circumstances (e.g., emergency hospital admissions), which suggests that some flexibility around session attendance could facilitate retention rates. A study delivering a lifestyle programme on T2D prevention in South Africa found that disruption of schedules is common because of family events, power outages, illnesses, or community protests.^22^ Another study in churches in rural settings in South Africa also found that challenges were faced in terms of scheduling of the sessions and programme attendance.^18^ While experiencing some similar challenges in terms of scheduling of sessions, overall retention in this study was high. Working more closely with member-led committees, groups and leadership at the places of worship can help with scheduling clashes. However, with a couples-based intervention, we would expect there to be difficulties accommodating two people’s schedules and we would make provisions within intervention delivery through recaps and handouts to allow those who have missed sessions to catch up with the information from sessions.

Delivering the Diabetes Together intervention in places of worship was found to be acceptable. Participants reported wanting more sessions, and facilitators’ feedback pointed to the plethora of questions arising during and after the sessions, indicating that participants might benefit from additional sessions. In this study, only four of the core modules of the intervention were delivered. The complete Diabetes Together intervention provides additional content on goal setting, substance use, sexual relationships, fears and complications, gender roles and stress management, which would help address participants’ questions. Although we did not measure self-management behaviour change in this study, participants reported gaining new knowledge, attitudes and skills that would support behaviour change. Researches on couples’ interventions for chronic disease management have shown that partners can be supported to positively influence and support self-management behaviour.^23,24^ In addition, taking part in such interventions can improve outcomes for partners, such as improvements in mood and reducing treatment burden or workload.^25^ These outcomes and mechanisms of action are in line with our logic model and we would measure changes in these variables in a future RCT.^12^

The study has several strengths. As far as we know, this is the first couples-based T2D intervention carried out in places of worship in South Africa. The findings of this research contribute to the body of literature around the feasibility of conducting health interventions in places of worship. The inclusion of a masjid extends existing literature that has pre-dominantly included interventions in church settings. A limitation was the use of English as the language of instruction and communication, because of time and resource constraints to translate materials into local languages. No participants were excluded because of language barriers, although it may have influenced screening and recruitment. For an RCT, recruitment and intervention materials should be translated into the local languages of each community and be sensitive to cultural norms. Another limitation was that participants who attend places of worship may be more motivated to improve their health or receptive to health messaging and this may not be reflective of the wider population. Reimbursing for the time spent by providing grocery vouchers as well as social desirability bias as participants belong to the same congregation, may have positively influenced retention during the intervention delivery period.

## Conclusion

Our findings show that places of worship are feasible settings for the delivery of the Diabetes Together intervention, with high levels of engagement. However, the recruitment target was not reached, suggesting that this approach could be included as part of a broader recruitment strategy for a randomised controlled efficacy trial in the future. As there is limited evidence on intervention delivery in places of worship in the community, we have made recommendations to support successful intervention delivery in these settings. These include undertaking formative work to engage and build partnership with the leadership, linking with satellite venues, approaching places with larger congregation sizes, collaborating closely with leadership and lay members to plan intervention delivery schedules, and ensuring that adequate resources are budgeted for outreach activities and flexible timelines. Future research on using places of worship to deliver interventions aimed at other chronic disease prevention and management programmes is warranted.
